# The Transcriptome of Paired Major and Minor Salivary Gland Tissue in Patients With Primary Sjögren’s Syndrome

**DOI:** 10.3389/fimmu.2021.681941

**Published:** 2021-07-06

**Authors:** Gwenny M. Verstappen, Lu Gao, Sarah Pringle, Erlin A. Haacke, Bert van der Vegt, Silvia C. Liefers, Vishal Patel, Yanhua Hu, Sumanta Mukherjee, Julie Carman, Laurence C. Menard, Frederik K. L. Spijkervet, Arjan Vissink, Hendrika Bootsma, Frans G. M. Kroese

**Affiliations:** ^1^ Rheumatology and Clinical Immunology, University Medical Center Groningen, University of Groningen, Groningen, Netherlands; ^2^ Immunology, Cardiovascular, Fibrosis Thematic Research Center, Translational Early Development, Bristol-Myers Squibb, Princeton, NJ, United States; ^3^ Pathology and Medical Biology, University Medical Center Groningen, University of Groningen, Groningen, Netherlands; ^4^ Oral and Maxillofacial Surgery, University Medical Center Groningen, University of Groningen, Groningen, Netherlands

**Keywords:** Sjogren’s syndrome, autoimmune disease, transcriptome (RNA-seq), B cell abnormalities, T cell activation, salivary gland

## Abstract

**Background:**

While all salivary glands (SGs) can be involved in primary Sjögren’s syndrome (pSS), their respective role in pathogenesis remains unclear. Our objective was to assess immunopathway activation in paired parotid and labial gland tissue from biopsy-positive and biopsy-negative pSS and non-SS sicca patients.

**Methods:**

Paraffin-embedded, paired parotid and labial salivary gland tissue and peripheral blood mononuclear cells were obtained from 39 pSS and 20 non-SS sicca patients. RNA was extracted, complementary DNA libraries were prepared and sequenced. For analysis of differentially expressed genes (DEGs), patients were subdivided based on fulfillment of ACR-EULAR criteria and histopathology.

**Results:**

With principal component analysis, only biopsy-positive pSS could be separated from non-SS sicca patients based on SG gene expression. When comparing the transcriptome of biopsy-positive pSS and biopsy-negative non-SS sicca patients, 1235 and 624 DEGs (FDR<0.05, log2FC<-1 or >1) were identified for parotid and labial glands, respectively. The number of DEGs between biopsy-negative pSS and non-SS sicca patients was scarce. Overall, transcript expression levels correlated strongly between parotid and labial glands (R^2^ = 0.86, p-value<0.0001). Gene signatures present in both glands of biopsy-positive pSS patients included IFN-α signaling, IL-12/IL-18 signaling, CD3/CD28 T-cell activation, CD40 signaling in B-cells, DN2 B-cells, and FcRL4+ B-cells. Signature scores varied considerably amongst pSS patients.

**Conclusion:**

Transcriptomes of paired major and minor SGs in pSS were overall comparable, although significant inter-individual heterogeneity in immunopathway activation existed. The SG transcriptome of biopsy-negative pSS was indistinguishable from non-SS sicca patients. Different patterns of SG immunopathway activation in pSS argue for personalized treatment approaches.

## Introduction

In primary Sjögren’s syndrome (pSS), salivary and lacrimal glands are considered main targets of the disease (1). A pathogenic hallmark of pSS is B-cell hyperactivity, reflected by the presence of autoantibodies (anti-SSA/Ro, anti-SSB/La, rheumatoid factor), hypergammaglobulinemia, and the increased risk of developing B-cell lymphoma (2). In addition to the presence of disease-associated autoantibodies, another important diagnostic feature of pSS is periductal focal lymphocytic sialadenitis (FLS) in salivary glands (SGs) (3). FLS with a focus score (FS) ≥1 indicates a positive SG biopsy (4). Other histologic features associated with pSS are the presence of lymphoepithelial lesions (LELs) and a plasma cell shift (relative decrease of IgA plasma cells with influx of IgM and/or IgG plasma cells). Furthermore, within the SG infiltrates, ectopic lymphoid structures can develop that may contain germinal centers (5). While the intimate interaction between glandular epithelium and immune cells is likely critical to pSS pathogenesis (6), a clear correlation between immune cell infiltration and main patient-reported symptoms (i.e. dryness, pain and fatigue) or salivary flow rates is lacking (7–9). There is, however, a positive association between FS and the amount of atrophy and fibrosis (8, 10), indicating that infiltrates can lead to glandular tissue damage.

SG infiltrates are mainly composed of CD4^+^ T-cells and B-cells, but may also harbor other immune cells, such as dendritic cells and natural killer cells (11). T-cell dependent B-cell hyperactivity and type-I interferon (IFN) activation are both thought to play central roles in the immunopathogenesis of pSS (2, 12, 13). Peripheral B-cell and type-I IFN activation signatures, measured by whole blood transcriptomics, have been associated with separate clinical phenotypes (14). Other ways to stratify pSS patients based on immune signatures in blood have also been proposed (15, 16). Alternatively, patient stratification could be based on immune signatures in SG tissue, although so far only smaller-sized transcriptomic studies with labial salivary gland (LSG) tissue have been performed (17–19). While all types of SGs can be involved in pSS, LSGs are most commonly biopsied for diagnostic purposes, with parotid salivary glands (PSGs) as a suitable alternative (20). The respective roles and contributions of different glands to immunopathogenesis remain unclear. Such knowledge would advance our understanding of the disease process and is essential for patient stratification and clinical trial design with targeted immunotherapies. We therefore analyzed the transcriptome of paired PSG and LSG tissue, as well as matched peripheral blood mononuclear cells (PBMCs) from pSS and non-SS sicca patients. We compared immune signatures between biopsy-positive and -negative patient groups and tissues and correlated our findings to clinical and histopathological parameters.

## Materials and Methods

### Study Population

We included 39 consecutive patients with pSS who participated in our inception cohort and fulfilled 2016 ACR-EULAR criteria (21). Twenty non-SS sicca patients from the same cohort, age- and sex-matched to the pSS group, were also included. Patients agreed to undergo both a parotid and labial gland biopsy. Matched cryopreserved PBMC, whole blood, and serum samples, collected at inclusion, were also available. Paired parotid and labial gland samples were always collected on the same day. Blood samples were collected when patients visited the rheumatologist around the same time (maximum of one month before or three months after the biopsy). No clinical interventions were initiated before biopsies were taken. Informed consent was obtained according to the Declaration of Helsinki. The study was approved by the Medical Research Ethics Committee of the University Medical Center Groningen (METc2013.066).

### Subgroup Analysis Based on Histopathology

We divided patients into four groups based on fulfillment of 2016 ACR-EULAR criteria and histopathological phenotype. The rational for this grouping was based on observations that non-SS sicca patients may have mild lymphocytic infiltration and pSS patients may have a negative biopsy result (i.e., focus score<1.0). For the purpose of this study, a positive biopsy was defined as FLS with focus score≥1 and/or presence of LELs, because LELs are highly specific for pSS compared with non-SS sicca (22). Biopsies with sclerosing chronic sialadenitis, mucosa-associated lymphoid tissue (MALT) lymphoma, or severe atrophy and PSG biopsies containing a lymph node were excluded.

### RNA Extraction and Sequencing Library Preparation

RNA was extracted from 11 formalin-fixed (4%), paraffin-embedded tissue sections (4μm) per biopsy. After scraping the tissue from the slides and deparaffinization, tissue was digested in buffer PKD (Qiagen, USA). RNA was extracted using the RNAeasy FFPE kit, according to manufacturer’s protocol (Qiagen, USA). For RNA extraction from PBMCs, 3x10^6 cells were thawed and rested for 1h in RPMI medium supplemented with 10%FBS and Antibiotic-Antimycotic (all from Gibco, UK) at 37°C. After resting, cell lysis and RNA isolation were performed using the RNAeasy Mini kit, according to manufacturer’s protocol (Qiagen, USA). RNA was immediately stored at -80°C until further use. Complementary DNA library preparation was performed by using TruSeq Stranded Total RNA Library Prep Gold (Illumina, USA, Cat#20020598), following manufacturer’s recommendations. Prepared libraries were sequenced on a HiSeq 2500 System (Illumina, USA).

### Transcriptomic Data Analysis

Data quality assessment and principal component analysis (PCA) were performed to understand the main source of variability. Differential expression analysis was performed using the limma package (v3.42.2) in R (v3.6.1) to identify differentially expressed genes (DEGs) across subgroups. Adjusted P-values were calculated using the Benjamini-Hochberg method; a gene was considered differentially expressed if the absolute fold change was >2 or <0.5 and the false discovery rate-adjusted P-value was <0.05. The *MetaCore* from *Clarivate Analytics* pathway database was used for pathway enrichment analysis. Additionally, gene signatures were established based on either published data (i.e., DN2 B-cell signature, FcRL4+ B-cell signature (23, 24)) or *in vitro* modulation of healthy human PBMCs or purified cells. Details on gene signature generation and composition are provided in **Supplementary Tables 1** and **2**. The single-sample Gene Set Enrichment Analysis algorithm was used to calculate the score of pre-defined gene signatures per sample (25). For evaluation of the correlation between average gene expression in the two types of salivary gland, only patients with paired samples were included.

### Peripheral Blood Cytokine Analysis

Concentrations of 84 cytokines in serum were assessed using multiplexed bead-based immunoassays (Merck Millipore, USA). Concentrations of myxovirus resistance protein A (MxA), a surrogate marker for type-I IFN activity (26), were measured in lysed whole blood using an in-house ELISA (see **Supplementary Methods**).

### Histological Assessments

For each biopsy, the focus score and area fraction of the infiltrate were calculated. To define the SG area occupied by the infiltrate, sections were immunohistochemically stained with anti-CD45 (Dako, clone 2B11+PD7/26). The relative amount of CD45-positive infiltrate was assessed in relation to the total amount of tissue (excluding larger areas of fat and fibrotic tissue) by a pixel-based digital image analysis in QuPath (27).

## Results

Patient characteristics are depicted in **Table 1**. Out of the 59 patients included in this study, one patient had withdrawn consent for the labial gland biopsy after inclusion.

**Table 1:** Demographic, clinical and histopathological characteristics of the study population.

Characteristic	pSS (n=39)	non-SS sicca (n=20)	P-value
Female sex, n (%)	38 (97)	19 (95)	–
Age in years, mean ± SD	52 ± 14	50 ± 14	0.573*
ESSDAI, median (range)	4 (0-29)	–	–
*- Cutaneous*, *n (%)*	*1 (3)*		
*- Respiratory*, *n (%)*	*4 (10)*		
*- Renal*, *n (%)*	*0 (0)*		
*- Articular*, *n (%)*	*13 (33)*		
*- Muscular*, *n (%)*	*0 (0)*		
*- PNS*, *n (%)*	*1 (3)*		
*- CNS*, *n (%)*	*0 (0)*		
*- Hematological*, *n (%)*	*13 (33)*		
*- Glandular*, *n (%)*	*17 (43)*		
*- Constitutional*, *n (%)*	*11 (28)*		
*- Lymphadenopathy*, *n (%)*	*4 (10)*		
*- Biological*, *n (%)*	*23 (58)*		
ESSPRI, median (range)	7.0 (1.3-8.7)	–	–
Xerostomia, n (%)	38 (97)	20 (100)	–
UWSF (mL/min.), median (range)	0.08 (0-0.68)	0.17 (0-0.58)	0.208*
ANA positive, n (%)	34 (85)	9 (45)	0.002**
ANA ≥1:80, n (%)†	25 (66)	2 (10)	<0.001**
Anti-SSA/Ro positive, n (%)	30 (77)	1 (5)	–
Anti-SSB/La positive, n (%)	17 (44)	0 (0)	–
RF positive, n (%)	25 (64)	0 (0)	–
IgG (g/L), mean ± SD	17.1 ± 5.5	9.9 ± 2.2	<0.001*
Focus score, median (range)			
*Labial gland*	1.5 (0.0-12)	0.4 (0.0-1.4)	<0.001*
*Parotid gland*	1.0 (0.0-12)	0.0 (0.0-1.7)	0.001*
Plasma cell shift, n (%)			
*Labial gland*	20 (53)	1 (5)	<0.001**
*Parotid gland*	14 (39)	0 (0)	0.001**
DMARD use, n (%)			
- *Hydroxychloroquine*	5 (13)	1 (5)	–
- *Methotrexate*	1 (3)	0 (0)	–
- *Azathioprine*	1 (3)	0 (0)	–
Corticosteroid use, n (%)	1 (3)	1 (5)	–

^†^For two pSS patients, the exact ANA titer was not available *Statistical test for continuous values. An Independent t Test or Mann-Whitney U Test was performed, based on the distribution of data; **Fisher’s exact test.

Of the remaining 58 patients with a paired PSG and LSG biopsy, we excluded seven SG samples (each from individual patients) for the following reasons: confirmed MALT lymphoma (n=1; parotid), sclerosing chronic sialadenitis (n=1; labial), presence of a lymph node (n=3; parotid), or severe atrophy (n=2; parotid) from the analysis. Thus, in total 52 PSG and 57 LSG samples were included in the analyses with 51 paired biopsies of 34 pSS and 17 non-SS sicca patients. Numbers of included samples per subgroup are shown in **Table 2**. PBMC, serum, and whole blood samples were available from 59, 57, and 52 patients, respectively. Because the biopsy-positive non-SS group, as expected, contained only few samples, this group was excluded from statistical analyses.

**Table 2:** Definition of subgroups based on histopathological phenotype.

Group	Fulfillment of ACR-EULAR criteria*	Positive SG biopsy**	*n* for parotid	*n* for labial
**I**	No	No	16	17
**II**	No	Yes	2	2
**III**	Yes	No	13	5
**IV**	Yes	Yes	21	33

*For fulfillment of the criteria, only one of the two biopsies (labial or parotid) needed to have a focus score ≥1. **Definition of positive biopsy in the current study: Focus score≥1 and/or presence of lymphoepithelial lesions.

### Only Biopsy-Positive pSS Patients Show an Altered SG Transcriptome

**Figure 1:**
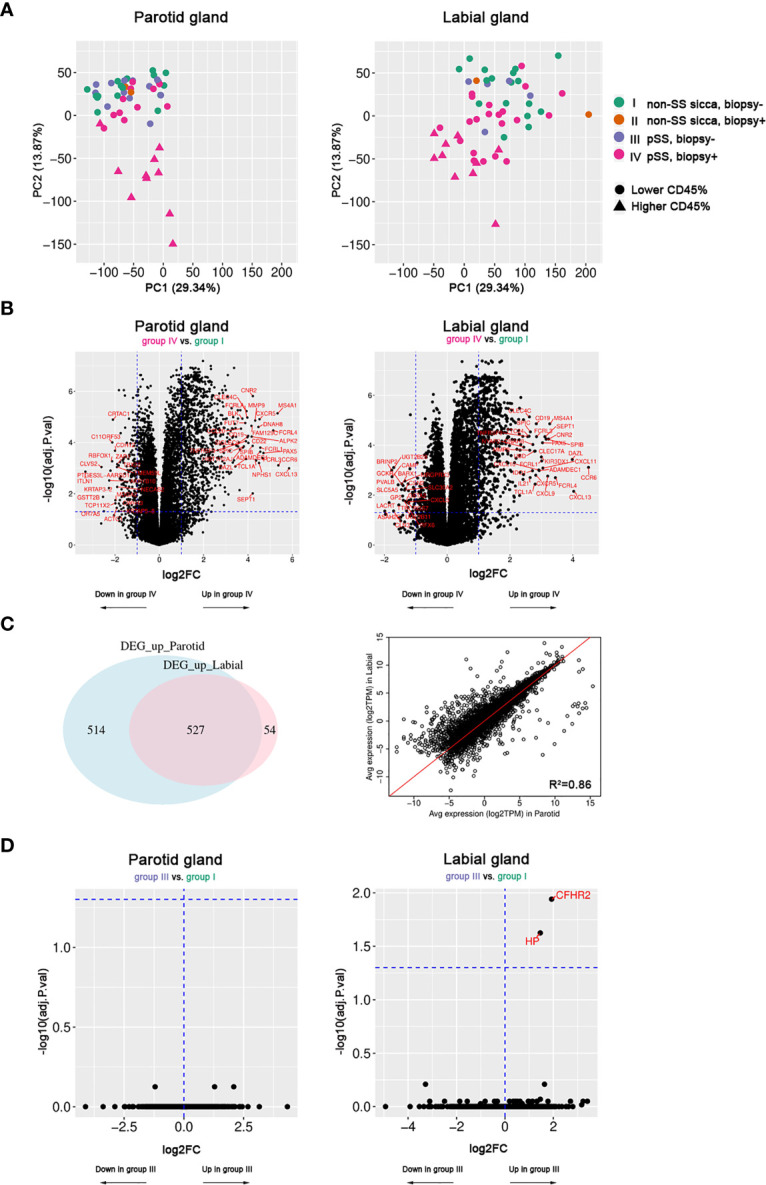
Transcriptome analysis of paired parotid and labial gland samples. Fifty-two parotid samples and 57 labial samples were included. **(A)** Principal component analysis of parotid and labial gland samples. Colors indicate the patient groups and triangles indicate the 10 samples with the highest area fraction of CD45^+^ cells. **(B)** Volcano plots showing differentially expressed genes (DEGs) between biopsy-positive pSS patients (group-IV) and biopsy-negative non-SS sicca patients (group-I) for parotid and labial gland samples. **(C)** Venn diagram of (overlapping) DEGs between parotid and labial gland samples and correlation plot for up- and down-regulated genes (adj.p<0.05, log2FC>1) in either tissue. **(D)** Volcano plots showing hardly any differential gene expression between biopsy-negative pSS patients (group-III) and biopsy-negative non-SS sicca patients (group-I) for parotid and labial gland samples.

First, we performed a PCA to explore whether PSG and LSG samples from pSS and non-SS sicca patients could be distinguished by their transcriptome. PCA revealed that only pSS patients with a positive biopsy (group-IV) could be separated from biopsy-negative pSS (group-III) and non-SS sicca patients (groups I&II) based on gene expression analysis in either PSG or LSG tissue (**Figure 1A**). Distinct clustering was associated with a relative increase in immune infiltrate size, measured by the CD45^+^ area of parenchymal tissue (**Figure 1A**).

Based on PCA results, we then compared the transcriptome of biopsy-positive pSS (group-IV) with biopsy-negative non-SS (group-I) to look for genes that were differentially expressed. For the PSG, 1041 up-regulated (FDR<0.05, log2FC>1) and 194 down-regulated (FDR<0.05, log2FC<-1) genes were identified. For the LSG, these numbers were 581 and 43, respectively. The top 20 up-regulated genes in both tissues were mostly B-cell or T-cell related (full list of DEGs in **Supplementary Tables 3** and **4**). Generally, large overlap between upregulated DEGs in PSGs and LSGs was observed (**Figures 1B, C**). There was a strong correlation between average gene expression in PSGs and LSGs (R^2^ = 0.86, **Figure 1C**) and between transcript expression fold changes (R^2^ = 0.59) when taking all genes into account. Notably, also the focus score and area fraction of CD45+ cells showed a good correlation between paired PSG and LSG samples from pSS patients (Spearman’s rho=0.65 and 0.66, respectively; p<0.001; **Supplementary Figure 1**), although higher values were observed in LSG *vs*. PSG tissue (**Table 1**). Despite a higher degree of infiltration in LSG tissue, we observed a tendency towards higher transcript fold changes in PSG tissue when comparing biopsy-positive pSS and biopsy-negative non-SS patients (**Supplementary Figure 2**). Genes with poorly correlated expression levels between the two SG types were not associated with immune function and mostly related to SG biology (e.g., genes encoding cystatins, ATPases, carbonic anhydrase VI, and α-amylase; data not shown).

Secondly, we compared the transcriptome of biopsy-positive pSS (group-IV) and biopsy-negative pSS (group-III) patients. DEGs were very similar to those identified in the previous comparison (group-IV *vs*. group-I; data not shown). In marked contrast, when we compared biopsy-negative pSS (group-III) with biopsy-negative non-SS sicca (group-I) patients, only two genes in LSG and none in PSG tissue were differentially regulated (**Figure 1D**). While transcriptomes of these groups were almost indistinguishable, at the functional level we noted some difference, since unstimulated whole salivary flow (UWSF) rates tended to be higher in group-I *vs*. group-III for the LSG (not PSG) comparison (p=0.13). There were no differences in stimulated WSF (SWSF) rates between the groups (**Supplementary Table 5**).

### A Key Set of Immunopathways Is Active in Both Glands

**Figure 2:**
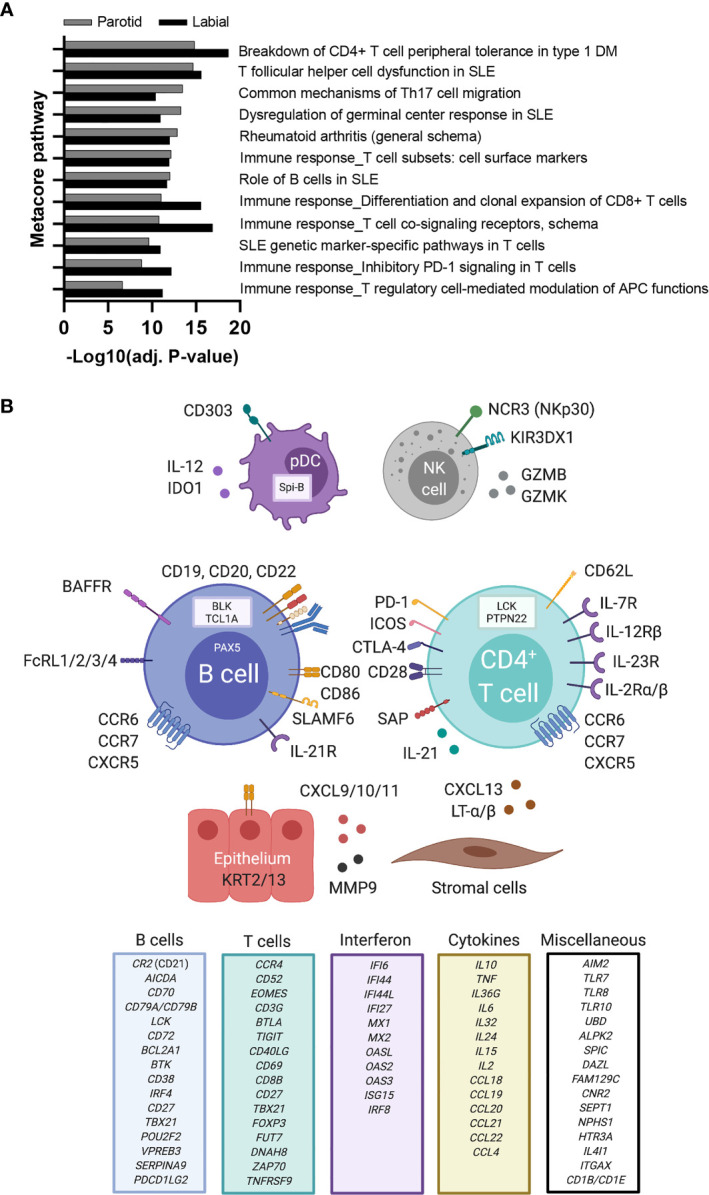
Upregulated immune pathways and immune-related genes in labial and parotid gland tissue of primary SS patients. **(A)** Metacore pathway analysis of differentially expressed genes (DEGs) between biopsy-positive primary SS and biopsy-negative non-SS sicca patients. A separate pathway analysis was performed for labial and parotid gland tissue. Only the top (auto)immune-related pathways that were enriched in both glands are shown (FDR<0.05). Enrichment values provided by Metacore are displayed as −Log(adj.p) for parotid gland tissue (grey bars) and labial gland tissue (white bars). **(B)** DEGs upregulated in salivary gland tissue of biopsy-positive pSS patients are depicted in association with their predicted cell of origin. Other DEGs with known immune function were categorized and are listed in separate boxes. Created with Biorender.com.

Next, we analyzed whether specific pathways were enriched amongst up-regulated genes in SGs of biopsy-positive pSS compared to non-SS sicca patients. A pathway database was used for pathway enrichment analysis of DEGs. As expected, the majority of upregulated pathways involved T-cells or B-cells (**Figure 2A**). Identified pathways were largely similar between PSG and LSG analyses. A schematic overview of upregulated genes with known immune function is provided as **Figure 2B**.

**Figure 3:**
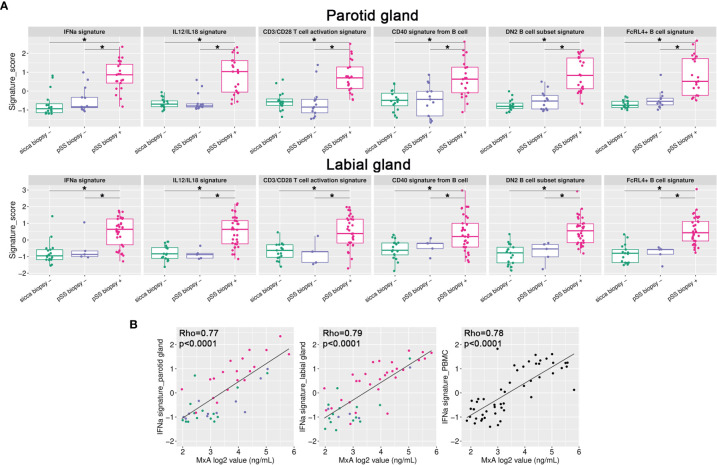
Gene signature analysis of paired salivary gland tissue samples. **(A)** Gene signature scores were calculated for all individual samples and plotted per signature per group for either the parotid or labial gland. *p < 0.005 **(B)** IFN-α signature scores in parotid and labial gland tissue and PBMCs were correlated with blood MxA levels. Colors represent the different patient groups as indicated in **Figure 1**.

Additionally, to obtain individual sample data on relevant biological processes, gene signature analysis was also performed. These signatures were selected based on current knowledge of immune pathways involved in pSS pathogenesis (12, 13, 28, 29). For both PSG and LSG tissue, the following signatures were significantly enriched in biopsy-positive pSS (group-IV) compared with either biopsy-negative pSS (group-III) or biopsy-negative non-SS sicca (group-I) patients: IFN-α signaling, IL-12/IL-18 signaling, CD3/CD28 T-cell activation, CD40 signaling in B-cells, double negative type-2 (DN2) B-cells, and FcRL4^+^ B-cells (**Figure 3A**). Within group-IV, signature scores varied considerably amongst individual patients. We also assessed other signatures, such as CD40 signaling in DCs or monocytes. Only for CD40 signaling in DCs, significantly higher scores were observed in labial glands (trend for parotid glands) of biopsy-positive pSS patients (group-IV *vs*. group-I; data not shown). We also observed enrichment of the TLR7 signature in group-IV, with a pattern similar to the IFN-α signaling signature (data not shown), probably attributed to the large overlap in genes that make up these two signatures. None of the signatures were significantly enriched in biopsy-negative pSS compared with non-SS sicca patients (**Figure 3A**).

### SG Gene Signatures Reflected in Blood

We also tested gene signature enrichment in matched PBMCs, to investigate whether transcriptional changes were shared between SGs and blood. Three PBMC samples were excluded after sequencing because of a high number of reads aligning to intronic regions, indicating genomic DNA contamination. When comparing biopsy-positive pSS with non-SS sicca patients, we found that the IFN-α signaling and DN2 B-cell signatures were also enriched in pSS patients’ PBMCs (**Supplementary Figure 3**). Upregulation of other gene signatures depends apparently on the tissue microenvironment. Importantly, IFN-α signature scores correlated significantly between blood and tissue (R^2^ = 0.64 and 0.70 for PSG and LSG, respectively). Not only the PBMC IFN-α signature score, but also blood levels of MxA protein correlated significantly with IFN-α signature scores in SG tissues (**Figure 3B**), indicating that blood MxA level is a biomarker for SG type-I IFN activity. SG IFN-α scores also significantly correlated with serum levels of two IFN-induced chemokines, i.e., CXCL10 and CXCL11 (**Supplementary Figure 4**), although not as strong as with blood MxA levels. The finding that blood MxA levels accurately reflect SG type-I IFN activity was also supported by the strong correlation between blood MxA and salivary gland *MX1* transcript expression levels (R^2^ = 0.66, p<0.0001 for parotid and R^2^ = 0.71, p<0.0001 for labial glands).

In addition to the IFN-α signature, we investigated other gene signatures elevated in SG tissue for their potential association with related serum cytokine levels. We found positive correlations between the SG DN2 B-cell score and serum CXCL13 levels, and for the CD3/CD28 T-cell activation score with serum CCL21 and sPD-1 levels (**Supplementary Figure 4**).

### SG Transcriptome and Autoantibody Positivity

**Figure 4:**
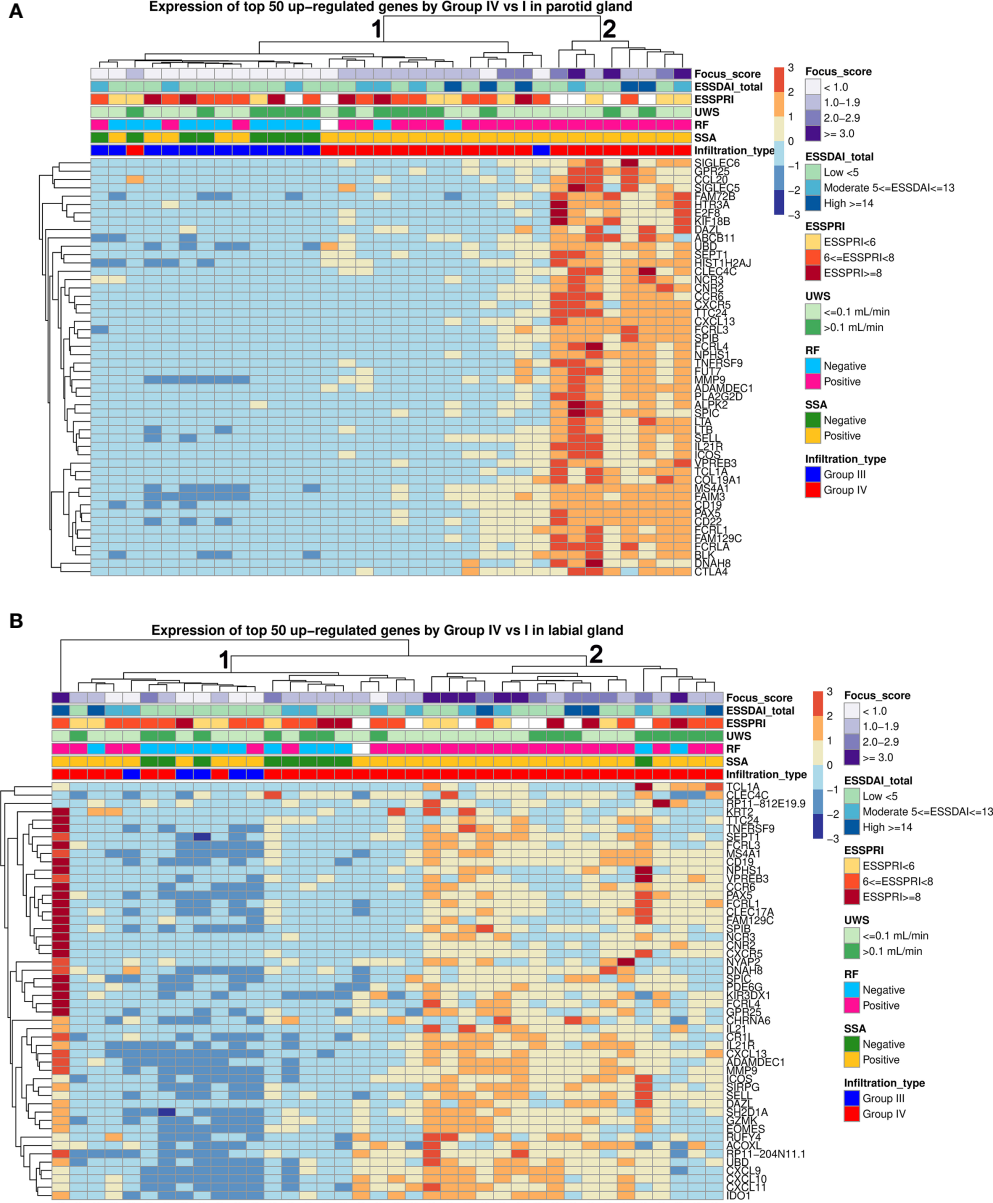
Hierarchical clustering of parotid and labial gland samples from pSS patients. Unsupervised hierarchical clustering for all pSS patients was performed based on expression of the top 50 upregulated genes in each tissue. Each column represents a patient and each row represents a gene. The following clinical parameters were categorized and marked: Diagnostic biopsy focus score, EULAR Sjögren’s syndrome disease activity index (ESSDAI), EULAR Sjögren’s syndrome patient reported index (ESSPRI), unstimulated whole saliva (UWS) flow rates, rheumatoid factor (RF) positivity, anti-SSA positivity, histopathological phenotype.

Unsupervised hierarchical clustering of pSS patients using the top-50 upregulated genes (group-IV *vs*. group-I) in either PSG or LSG tissue showed two main clusters: Cluster 1 with weak-to-moderate upregulation of top-50 DEGs and Cluster 2 with strong upregulation of these DEGs (**Figure 4**). Nearly all PSG- or LSG-defined Cluster 2 patients were anti-SSA and rheumatoid factor positive (**Figures 4A, B**). Patients with FS≥3.0 in either gland were all contained within this cluster. One biopsy-positive pSS patient clustered separately from other patients based on the LSG transcriptome (**Figure 4B**). In this patient, 91% of the LSG biopsy consisted of CD45^+^ cells. This patient had type-III cryoglobulinemia and MALT-lymphoma in the parotid gland. Schirmer’s test, UWSF and SWSF rates were all zero, reflecting the severe damage to the glands of this patient.

Lastly, we explored whether transcriptomic clusters correlated with clinical disease parameters. No significant differences in ESSDAI, ESSPRI, ESSPRI dryness, UWSF or SWSF was observed between the two clusters nor between autoantibody-positive and -negative pSS patients.

## Discussion

Transcriptomic analysis of paired major and minor SG biopsies of pSS patients revealed a high degree of overlap in immune pathway activity. Only tissue samples from biopsy-positive pSS were distinct from non-SS sicca patients based on the SG transcriptome. This distinction was mainly driven by infiltrating immune cells. Between biopsy-negative pSS and non-SS sicca patients, we expected to find evidence for distinct mechanisms of (intrinsic) epithelial cell dysregulation, yet the SG transcriptome of both glands was strikingly similar between these groups. For biopsy-positive pSS patients we observed that immune pathways significantly upregulated in both glands were mostly related to CD4^+^ T-cells and B-cells. The IFN-α gene signature was also significantly enriched in these patients and a strong correlation between the IFN-α score in PBMCs and SGs was seen, indicating that SG type-I IFN activity is reflected systemically. Our results further show that blood MxA level is a surrogate biomarker of type-I IFN activity in SGs.

We observed a strong correlation between average gene expression in PSGs and LSGs. The number of DEGs and transcript expression fold changes between biopsy-positive pSS and non-SS sicca patients were, however, higher for PSGs. This may be explained by the higher amount of infiltration in LSG *vs*. PSG tissue of non-SS sicca patients. Notably, in pSS patients the LSG biopsy was more often classified as positive than PSG. LSGs may be more likely to harbor non-specific infiltration, as lymphocytic foci are present in approximately 15% of healthy individuals (30, 31), while for PSGs this is estimated at 5% (20). Despite these differences, Metacore pathway analysis showed that all top-20 enriched (auto)immune pathways were shared between the two different SG tissues. Importantly, immunopathways and DEGs identified in our study correspond largely to those presented in a recent transcriptomic study of LSG tissue in pSS (17).

Although many DEGs were identified in biopsy-positive pSS compared to biopsy-negative non-SS sicca patients, we found that biopsy-negative pSS patients did not have an altered gene expression profile in their PSGs and LSGs, compared to non-SS sicca patients. While not directly comparing PSGs with LSGs, we noted that genes with poorly correlated expression levels between glands were all related to SG biology, not immunopathology. Although there are indications that intrinsic epithelial cell abnormalities contribute to SG disease in pSS (32), our findings do not support this hypothesis, at least not at the transcriptomic level. It might be that our approach does not capture subtle differences between epithelial cells of biopsy-negative pSS and non-SS sicca patients, or that non-SS sicca patients also exhibit epithelial abnormalities. Lastly, we cannot exclude that some non-SS sicca patients are in an early, pre-clinical stage of pSS, although the progression rate from non-SS sicca to pSS is estimated at only 10% over 2-3 years (33).

For biopsy-positive pSS, our study confirms the relevance of multiple SG immunopathways that have been previously published using other experimental approaches (34–38), although previous approaches were often focused on a single pathway and unable to measure activity of multiple pathways at the individual patient level. In the current study, upregulated gene signatures in both PSGs and LSGs of biopsy-positive pSS patients concerned IFN-α (and TLR7) signaling, IL-12/IL-18 signaling, CD3/CD28 T-cell activation, CD40 signaling in B-cells, DN2 B-cells, and FcRL4^+^ B-cells, with heterogeneity in signature scores amongst these patients. The signatures reveal that particularly B- and T-cells are activated, and these cells may exert pathogenic functions in the SGs. A type-I IFN signature is frequently observed in pSS patients, both in blood and SGs (26, 34, 39). This signature is a consequence of type-I IFN production in SGs, most likely by epithelial cells and plasmacytoid dendritic cells (37, 40). IFN-α production is induced in response to (viral) RNA sensing mainly through TLR7/8 and cytosolic sensors (13). We did not find evidence for the presence of an IFN-α signature in SGs of biopsy-negative pSS patients, suggesting that this signature is tightly correlated with the amount of infiltrate, in line with literature (34). Another study showed that SS patients with FS<1.0 were less likely to have elevated expression of IFN-regulated genes in PBMCs than anti-SSA-positive SS patients with FS≥1.0 (41). We further show that there is a strong correlation between IFN-α signature scores in paired major and minor SGs and PBMCs samples. Ongoing production of type-I and other types of IFNs could maintain a pro-inflammatory state by activating immune cells and stimulating cytokine production (e.g., CXCL10, BAFF) by epithelial cells, thereby negatively affecting SG homeostasis (37, 40, 42, 43). IFNs may also play a role in glandular T-cell activation by stimulating IL-7 production in epithelial cells (44). In our study, T-cell activation in SG lesions was reflected by an increase in the CD3/CD28 T-cell activation score and enrichment of T-cell related pathways, with Tfh-cell dysfunction at the top. Enrichment of Tfh-cells in pSS-derived SG tissue was recently demonstrated and associated with the presence of ectopic lymphoid structures (35, 45). Another gene signature enriched in SG tissue was IL-12/IL-18 signaling. IL-12, but not IL-18, was upregulated in both PSG and LSG tissue and could drive Th1-cell differentiation and type-II IFN production *via* STAT4 phosphorylation (46). Polymorphisms in both *IL12A* and *STAT4* genes have been associated with pSS and patients carrying the *IL12A* risk allele had increased IL-12p70 serum levels (29, 47).

While multiple immune cells are involved in the inflammatory response, the top-20 upregulated genes in PSG and LSG tissue of biopsy-positive pSS were mostly related to B-cells, underpinning the central role of B-cell hyperactivity in pSS pathogenesis. Combined SG microarray datasets also have shown that key driver genes in SS patients with high-grade SG inflammation were mostly related to BCR signaling and B-cell activation (19). By using SG gene signature analysis, we showed that gene signatures of activated B-cell subsets were enriched, including DN2 B-cells and FcRL4^+^ B-cells. Upon TLR7 stimulation, DN2 B-cells participate in extrafollicular responses in autoimmune disease patients (23). Presence of ductal epithelium-associated FcRL4^+^ B-cells in SGs of pSS patients was previously demonstrated (48). These cells are proliferating and phenotypically related to DN2 B-cells (24, 48). We also observed increased scores for the CD40 signaling in B-cells signature in biopsy-positive pSS, in line with a recent RNA sequencing study of LSG B-cells (37). Ligation of CD40 by CD40L-expressing activated T-cells is essential for T-cell dependent B-cell activation. In pSS, chronic B-cell activation in SGs may exacerbate disease and eventually result in development of B-cell lymphoma (49).

Our results suggest that immunotherapy directed at T-cell dependent B-cell hyperactivity is particularly beneficial for biopsy-positive pSS patients. In previous studies, a positive SG biopsy has been associated with autoantibody positivity, hypergammaglobulinemia, corneal and conjunctival damage, and reduced UWSF rates (7). Furthermore, severe SG inflammation (FS≥3) has been associated with higher ESSDAI scores and higher risk of developing B-cell lymphoma (50). A positive biopsy correlates, however, poorly with symptoms of dry mouth or eyes (7), corroborating with our results that ESSPRI dryness scores were similar between transcriptomic clusters. We neither observed differences in other clinical parameters (e.g., ESSDAI, UWSF) between these clusters, emphasizing the complex relationship between local inflammation and clinical parameters assessed cross-sectionally. An apparent discrepancy between biological activity and patient-reported dryness symptoms in pSS has also been noted in several clinical trials (51–54), and may be partly attributed to symptom adaptation. Trial design is further complicated by clinical and biological patient heterogeneity (55). Our results underscore our limited understanding of SG hypofunction in absence of infiltrates and question whether biopsy-negative and biopsy–positive pSS patients should receive similar treatment. For biopsy-positive pSS, our study shows enrichment of distinct gene signatures that can be therapeutically targeted, although heterogeneous expression patterns existed amongst individuals. Therefore, tissue transcriptomics may form a valuable tool for establishing precision drug therapy in pSS.

## Data Availability Statement

The salivary gland RNA sequencing datasets presented in this study can be found in online repositories. The names of the repository/repositories and accession number(s) can be found below: https://www.ncbi.nlm.nih.gov/geo/query/acc.cgi?acc=GSE173808.

## Ethics Statement

The studies involving human participants were reviewed and approved by Medical Research Ethics Committee of the University Medical Center Groningen (METc 2013.066). The patients/participants provided their written informed consent to participate in this study.

## Author Contributions

GV, LG, SP, AV, HB, and FK contributed to conception and design of the study. AV and HB recruited the patients. GV, EH, BV, SC, VP, and FS contributed to patient material collection and/or data acquisition. LG and SH performed data quality assessment, data processing and the statistical analysis. GV, LG, SP, SM, JC, LM, and FK were involved in data analysis and interpretation of results. GV wrote the first draft of the manuscript. LG wrote sections of the manuscript. All authors contributed to the article and approved the submitted version.

## Funding

This study was funded by Bristol-Myers Squibb (BMS). The funder was not involved in the study design, collection, analysis, interpretation of data, the writing of this article or the decision to submit it for publication. The work of GV, SP, HB, and FK is supported by a Dutch Arthritis Society (ReumaNL) Long Term Project Grant (LLP-29).

## Conflict of Interest

LG, YH, and LM are employed at BMS. VP is past employee of BMS and currently employed at Novartis. SM is past employee of BMS and currently employed at GlaxoSmithKline. JC is past employee of BMS and currently employed at Johnson&Johnson. BV is scientific advisory board member of Visiopharm. HB received unrestricted grants from BMS and Roche, is consultant for BMS, Roche, Novartis, Medimmune, Union Chimique Belge, speaker for BMS and Novartis. FK received unrestricted grants from BMS, is consultant for BMS, speaker for BMS, Roche and Janssen-Cilag.

The remaining authors declare that the research was conducted in the absence of any commercial or financial relationships that could be construed as a potential conflict of interest.

## References

[1] Brito-Zerón P Baldini C Bootsma H Bowman SJ Jonsson R Mariette X. Sjögren Syndrome. Nat Rev Dis Prim (2016) 2:16047. doi: 10.1038/nrdp.2016.47 27383445

[2] Kroese FG Abdulahad WH Haacke E Bos NA Vissink A Bootsma H. B-Cell Hyperactivity in Primary Sjogren’s Syndrome. Expert Rev Clin Immunol (2014) 10:483–99. doi: 10.1586/1744666X.2014.891439 24564507

[3] Shiboski CH Shiboski SC Seror R Criswell LA Labetoulle M Lietman TM. American College of Rheumatology/European League Against Rheumatism Classification Criteria for Primary Sjögren’s Syndrome: A Consensus and Data-Driven Methodology Involving Three International Patient Cohorts. Arthritis Rheumatol (2017) 69:35–45. doi: 10.1002/art.39859 27785888 PMC5650478

[4] Greenspan JS Daniels TE Talal N Sylvester RA. The Histopathology of Sjögren’s Syndrome in Labial Salivary Gland Biopsies. Oral Surgery Oral Med Oral Pathol (1974) 37:217–29. doi: 10.1016/0030-4220(74)90417-4 4589360

[5] Kroese FGM Haacke EA Bombardieri M. The Role of Salivary Gland Histopathology in Primary Sjögren’s Syndrome: Promises and Pitfalls - PubMed. Clin Exp Rheum (2018) 36:222–33. 30156550

[6] Verstappen GM Pringle S Bootsma H Kroese FGM. Epithelial– Immune Cell Interplay in Primary Sjögren Syndrome Salivary Gland Pathogenesis. Nat Rev Rheumatol (2021) 1–16. doi: 10.1038/s41584-021-00605-2 33911236 PMC8081003

[7] Daniels TE Cox D Shiboski CH Schiødt M Wu A Lanfranchi H. Associations Between Salivary Gland Histopathologic Diagnoses and Phenotypic Features of Sjögren’s Syndrome Among 1,726 Registry Participants. Arthritis Rheum (2011) 63:2021–30. doi: 10.1002/art.30381 PMC3128201 21480190

[8] Bookman AAM Shen H Cook RJ Bailey D McComb RJ Rutka JA. Whole Stimulated Salivary Flow: Correlation With the Pathology of Inflammation and Damage in Minor Salivary Gland Biopsy Specimens From Patients With Primary Sjögren’s Syndrome But Not Patients With Sicca. Arthritis Rheum (2011) 63:2014–20. doi: 10.1002/art.30295 21337320

[9] Mossel E Delli K Van Nimwegen JF Stel AJ Kroese FGM Spijkervet FKL. Ultrasonography of Major Salivary Glands Compared With Parotid and Labial Gland Biopsy and Classification Criteria in Patients With Clinically Suspected Primary Sjögren’s Syndrome. Ann Rheum Dis (2017) 76:1883–9. doi: 10.1136/annrheumdis-2017-211250 28754802

[10] Leehan KM Pezant NP Rasmussen A Grundahl K Moore JS Radfar L. Minor Salivary Gland Fibrosis in Sjögren’s Syndrome is Elevated, Associated With Focus Score and Not Solely a Consequence of Aging. Clin Exp Rheumatol (2018) 36:S80–8. PMC5913007 29148407

[11] Christodoulou MI Kapsogeorgou EK Moutsopoulos HM. Characteristics of the Minor Salivary Gland Infiltrates in Sjogren’s Syndrome. J Autoimmun (2010) 34:400–7. doi: 10.1016/j.jaut.2009.10.004 19889514

[12] Nocturne G Mariette X. B Cells in the Pathogenesis of Primary Sjögren Syndrome. Nat Rev Rheumatol (2018) 14:133–45. doi: 10.1038/nrrheum.2018.1 29416129

[13] Bodewes ILA Björk A Versnel MA Wahren-Herlenius M. Innate Immunity and Interferons in the Pathogenesis of Sjögren’s Syndrome. Rheumatol (Oxford) (2019) key360. doi: 10.1093/rheumatology/key360 30770713

[14] Tarn JR Howard-Tripp N Lendrem DW Mariette X Saraux A Devauchelle-Pensec V. Symptom-Based Stratification of Patients With Primary Sjögren’s Syndrome: Multi-Dimensional Characterisation of International Observational Cohorts and Reanalyses of Randomised Clinical Trials. Lancet Rheumatol (2019) 1:e85–94. doi: 10.1016/s2665-9913(19)30042-6 PMC7134527 38229348

[15] Mingueneau M Boudaoud S Haskett S Reynolds TL Nocturne G Norton E. Cytometry by Time-of-Flight Immunophenotyping Identifies a Blood Sjögren’s Signature Correlating With Disease Activity and Glandular Inflammation. J Allergy Clin Immunol (2016) 137:1809–1821.e12. doi: 10.1016/j.jaci.2016.01.024 27045581

[16] James JA Guthridge JM Chen H Lu R Bourn RL Bean K. Unique Sjögren’s Syndrome Patient Subsets Defined by Molecular Features. Rheumatol (Oxford) (2020) 59:860–8. doi: 10.1093/rheumatology/kez335 PMC7188221 31497844

[17] Oyelakin A Horeth E Song E-AC Min S Che M Marzullo B. Transcriptomic and Network Analysis of Minor Salivary Glands of Patients With Primary Sjögren’s Syndrome. Front Immunol (2021) 11:606268. doi: 10.3389/fimmu.2020.606268 33488608 PMC7821166

[18] Liu Z Li F Pan A Xue H Jiang S Zhu C. Elevated CCL19/CCR7 Expression During the Disease Process of Primary Sjögren’s Syndrome. Front Immunol (2019) 10:795. doi: 10.3389/fimmu.2019.00795 31068931 PMC6491632

[19] Min HK Moon SJ Park KS Kim KJ. Integrated Systems Analysis of Salivary Gland Transcriptomics Reveals Key Molecular Networks in Sjögren’s Syndrome. Arthritis Res Ther (2019) 21:294. doi: 10.1186/s13075-019-2082-9 31856901 PMC6921432

[20] Pijpe J Kalk WW van der Wal JE Vissink A Kluin PM Roodenburg JL. Parotid Gland Biopsy Compared With Labial Biopsy in the Diagnosis of Patients With Primary Sjogren’s Syndrome. Rheumatol (Oxford) (2007) 46:335–41. doi: 10.1093/rheumatology/kel266 16891656

[21] van Nimwegen JF van Ginkel MS Arends S Haacke EA van der Vegt B Sillevis Smitt-Kamminga N. Validation of the ACR-EULAR Criteria for Primary Sjögren’s Syndrome in a Dutch Prospective Diagnostic Cohort. Rheumatol (Oxford) (2018) 57:818–25. doi: 10.1093/rheumatology/kex495 29444331

[22] van Ginkel MS Haacke EA Bootsma H Arends S van Nimwegen JF Verstappen GM. Presence of Intraepithelial B-Lymphocytes is Associated With the Formation of Lymphoepithelial Lesions in Salivary Glands of Primary Sjögren’s Syndrome Patients. Clin Exp Rheumatol (2019) 37:S42–8. doi: 10.1136/annrheumdis-2018-eular.6637 31074726

[23] Jenks SA Cashman KS Zumaquero E Marigorta UM Patel AV Wang X. Distinct Effector B Cells Induced by Unregulated Toll-Like Receptor 7 Contribute to Pathogenic Responses in Systemic Lupus Erythematosus. Immunity (2018) 49:725–39. doi: 10.1016/j.immuni.2018.08.015 PMC6217820 30314758

[24] Verstappen GM Ice JA Bootsma H Pringle S Haacke EA de Lange K. Gene Expression Profiling of Epithelium-Associated FcRL4+ B Cells in Primary Sjögren’s Syndrome Reveals a Pathogenic Signature. J Autoimmun (2020) 109:102439. doi: 10.1016/j.jaut.2020.102439 32201227 PMC7337041

[25] Barbie DA Tamayo P Boehm JS Kim SY Moody SE Dunn IF. Systematic RNA Interference Reveals That Oncogenic KRAS-Driven Cancers Require TBK1. Nature (2009) 462:108–12. doi: 10.1038/nature08460 PMC2783335 19847166

[26] Maria NI Brkic Z Waris M van Helden-Meeuwsen CG Heezen K van de Merwe JP. Versnel M a. MxA as a Clinically Applicable Biomarker for Identifying Systemic Interferon Type I in Primary Sjogren’s Syndrome. Ann Rheum Dis (2014) 73:1052–9. doi: 10.1136/annrheumdis-2012-202552 PMC4033122 23831963

[27] Bankhead P Loughrey MB Fernández JA Dombrowski Y McArt DG Dunne PD. QuPath: Open Source Software for Digital Pathology Image Analysis. Sci Rep (2017) 7:1–7. doi: 10.1038/s41598-017-17204-5 29203879 PMC5715110

[28] Verstappen GM Kroese FGM Bootsma H. T Cells in Primary Sjögren’s Syndrome: Targets for Early Intervention. Rheumatol (Oxford) (2019) kez004. doi: 10.1093/rheumatology/kez004 PMC8516500 30770920

[29] Fogel O Rivière E Seror R Nocturne G Boudaoud S Ly B. Role of the IL-12/IL-35 Balance in Patients With Sjögren Syndrome. J Allergy Clin Immunol (2018) 142:258–268.e5. doi: 10.1016/j.jaci.2017.07.041 28916184

[30] Radfar L Kleiner DE Fox PC Pillemer SR. Prevalence and Clinical Significance of Lymphocytic Foci in Minor Salivary Glands of Healthy Volunteers. Arthritis Care Res (2002) 47:520–4. doi: 10.1002/art.10668 12382301

[31] Segerberg-Konttinen M. A Postmortem Study of Focal Adenitis in Salivary and Lacrimal Glands. J Autoimmun (1989) 2:553–8. doi: 10.1016/0896-8411(89)90188-1 2789657

[32] Manoussakis MN Kapsogeorgou EK. The Role of Intrinsic Epithelial Activation in the Pathogenesis of Sjogren’s Syndrome. J Autoimmun (2010) 35:219–24. doi: 10.1016/j.jaut.2010.06.011 20685080

[33] Shiboski CH Baer AN Shiboski SC Lam M Challacombe S Lanfranchi HE. Natural History and Predictors of Progression to Sjögren’s Syndrome Among Participants of the Sjögren’s International Collaborative Clinical Alliance Registry. Arthritis Care Res (2018) 70:284–94. doi: 10.1002/acr.23264 PMC5654699 28437595

[34] Hall JC Baer AN Shah AA Criswell LA Shiboski CH Rosen A. Molecular Subsetting of Interferon Pathways in Sjögren’s Syndrome. Arthritis Rheumatol (2015) 67:2437–46. doi: 10.1002/art.39204 PMC4551661 25988820

[35] Joachims ML Leehan KM Dozmorov MG Georgescu C Pan Z Lawrence C. Sjögren’s Syndrome Minor Salivary Gland CD4+ Memory T Cells Associate With Glandular Disease Features and Have a Germinal Center T Follicular Helper Transcriptional Profile. J Clin Med (2020) 9:2164. doi: 10.3390/jcm9072164 PMC7408878 32650575

[36] Pontarini E Murray-Brown WJ Croia C Lucchesi D Conway J Rivellese F. Unique Expansion of IL-21+ Tfh and Tph Cells Under Control of ICOS Identifies Sjögren’s Syndrome With Ectopic Germinal Centres and MALT Lymphoma. Ann Rheum Dis (2020) 79:1588–99. doi: 10.1136/annrheumdis-2020-217646 PMC7677495 32963045

[37] Rivière E Pascaud J Tchitchek N Boudaoud S Paoletti A Ly B. Salivary Gland Epithelial Cells From Patients With Sjögren’s Syndrome Induce B-Lymphocyte Survival and Activation. Ann Rheum Dis (2020) 79:1468–77. doi: 10.1136/annrheumdis-2019-216588 32843324

[38] Bombardieri M Barone F Pittoni V Alessandri C Conigliaro P Blades MC. Increased Circulating Levels and Salivary Gland Expression of Interleukin-18 in Patients With Sjögren’s Syndrome: Relationship With Autoantibody Production and Lymphoid Organization of the Periductal Inflammatory Infiltrate. Arthritis Res Ther (2004) 6:R447–56. doi: 10.1186/ar1209 PMC546280 15380044

[39] Nezos A Gravani F Tassidou A Kapsogeorgou EK Voulgarelis M Koutsilieris M. Type I and II Interferon Signatures in Sjogren’s Syndrome Pathogenesis: Contributions in Distinct Clinical Phenotypes and Sjogren’s Related Lymphomagenesis. J Autoimmun (2015) 63:47–58. doi: 10.1016/j.jaut.2015.07.002 26183766 PMC4564326

[40] Gottenberg JE Cagnard N Lucchesi C Letourneur F Mistou S Lazure T. Activation of IFN Pathways and Plasmacytoid Dendritic Cell Recruitment in Target Organs of Primary Sjogren’s Syndrome. Proc Natl Acad Sci U S A (2006) 103:2770–5. doi: 10.1073/pnas.0510837103 PMC1413808 16477017

[41] Sharma R Chaudhari KS Kurien BT Grundahl K Radfar L Lewis DM. Sjögren Syndrome Without Focal Lymphocytic Infiltration of the Salivary Glands. J Rheumatol (2020) 47:394–9. doi: 10.3899/jrheum.181443 PMC7304293 31092717

[42] Ittah M Miceli-Richard C Eric Gottenberg J Lavie F Lazure T Ba N. B Cell-Activating Factor of the Tumor Necrosis Factor Family (BAFF) is Expressed Under Stimulation by Interferon in Salivary Gland Epithelial Cells in Primary Sjogren’s Syndrome. Arthritis Res Ther (2006) 8:R51. doi: 10.1186/ar1912 16507175 PMC1526588

[43] Ogawa N Ping L Zhenjun L Takada Y Sugai S. Involvement of the Interferon-Gamma-Induced T Cell-Attracting Chemokines, Interferon-Gamma-Inducible 10-Kd Protein (CXCL10) and Monokine Induced by Interferon-Gamma (CXCL9), in the Salivary Gland Lesions of Patients With Sjögren’s Syndrome. Arthritis Rheum (2002) 46:2730–41. doi: 10.1002/art.10577 12384933

[44] Rivière E Pascaud J Virone A Dupré A Ly B Paoletti A. Interleukin-7/Interferon Axis Drives T-Cell and Salivary Gland Epithelial Cell Interactions in Sjögren’s Syndrome. Arthritis Rheumatol (2021) 73:631–40. doi: 10.1002/art.41558 33058491

[45] Pontarini E Verstappen GM Grigoriadou S Kroese FGM Bootsma H Bombardieri M. Blocking T Cell Co-Stimulation in Primary Sjögren’s Syndrome: Rationale, Clinical Efficacy and Modulation of Peripheral and Salivary Gland Biomarkers. Clin Exp Rheumatol (2020) 38(Suppl 126):222–7. 33095146

[46] Morinobu A Gadina M Strober W Visconti R Fornace A Montagna C. STAT4 Serine Phosphorylation is Critical for IL-12-Induced IFN-γ Production But Not for Cell Proliferation. Proc Natl Acad Sci U S A (2002) 99:12281–6. doi: 10.1073/pnas.182618999 PMC129436 12213961

[47] Nocturne G Boudaoud S Miceli-Richard C Viengchareun S Lazure T Nititham J. Germline and Somatic Genetic Variations of TNFAIP3 in Lymphoma Complicating Primary Sjögren’s Syndrome. Blood (2013) 122:4068–76. doi: 10.1182/blood-2013-05-503383 PMC3862283 24159176

[48] Haacke EA Bootsma H Spijkervet FKL Visser A Vissink A Kluin PM. FcRL4+ B-Cells in Salivary Glands of Primary Sjögren’s Syndrome Patients. J Autoimmun (2017) 81:90–8. doi: 10.1016/j.jaut.2017.03.012 28390747

[49] Nocturne G Mariette X. Sjogren Syndrome-Associated Lymphomas: An Update on Pathogenesis and Management. Br J Haematol (2015) 168:317–27. doi: 10.1111/bjh.13192 25316606

[50] Risselada AP Looije MF Kruize AA Bijlsma JW van Roon JA. The Role of Ectopic Germinal Centers in the Immunopathology of Primary Sjogren’s Syndrome: A Systematic Review. Semin Arthritis Rheum (2013) 42:368–76. doi: 10.1016/j.semarthrit.2012.07.003 22995442

[51] van Nimwegen JF Mossel E van Zuiden GS Wijnsma RF Delli K Stel AJ. Abatacept Treatment for Patients With Early Active Primary Sjögren’s Syndrome: A Single-Centre, Randomised, Double-Blind, Placebo-Controlled, Phase 3 Trial (ASAP-III Study). Lancet Rheumatol (2020) 2:e153–63. doi: 10.1016/S2665-9913(19)30160-2 38263653

[52] Baer AN Gottenberg JE St Clair EW Sumida T Takeuchi T Seror R. Efficacy and Safety of Abatacept in Active Primary Sjögren’s Syndrome: Results of a Phase III, Randomised, Placebo-Controlled Trial. Ann Rheum Dis (2020) 80:339–48. doi: 10.1136/annrheumdis-2020-218599 PMC7892395 33168545

[53] Fisher BA Szanto A Ng WF Bombardieri M Posch MG Papas AS. Assessment of the Anti-CD40 Antibody Iscalimab in Patients With Primary Sjögren’s Syndrome: A Multicentre, Randomised, Double-Blind, Placebo-Controlled, Proof-of-Concept Study. Lancet Rheumatol (2020) 2:e142–52. doi: 10.1016/S2665-9913(19)30135-3 38263652

[54] van der Heijden EHM Blokland SLM Hillen MR Lopes APP van Vliet-Moret FM Rosenberg AJWP. Leflunomide–hydroxychloroquine Combination Therapy in Patients With Primary Sjögren’s Syndrome (RepurpSS-I): A Placebo-Controlled, Double-Blinded, Randomised Clinical Trial. Lancet Rheumatol (2020) 2:e260–9. doi: 10.1016/S2665-9913(20)30057-6 38273473

[55] Verstappen GM Kroese FGM Bootsma H. Stumbles in Sjögren’s Syndrome Drug Development: Where to Look for the Next Big Leap? Expert Rev Clin Immunol (2020) 16:1043–5. doi: 10.1080/1744666X.2021.1831915 33196342

