# Supplementary material for: The Transcriptome of Paired Major and Minor Salivary Gland Tissue in Patients With Primary Sjögren’s Syndrome
Source: Front Immunol. 2021 Jul 6;12:681941. doi: 10.3389/fimmu.2021.681941 (PMC8291032; doi:10.3389/fimmu.2021.681941)
Supplement: Supplementary file 1 [file DataSheet_1.zip › Supplementary Methods_20210629.docx]

**Supplementary Methods**

*MxA ELISA*

One mL of heparinized, whole blood (stored at -80°C) was thawed and diluted 1:5 in lysis buffer (1.5% bovine serum albumin, 1% ascorbic acid, 0.5% NaHCO_3_ and 0.05% NaN_3_ in demi-H_2_O). Microtiter 96-well high binding plates (Costar) were coated with 100 μL of capture antibody (1.5 μg/mL rat anti-MxA mAb (clone 2D12; Biogen, USA) in 0.2 M sodium-carbonate-bicarbonate buffer, pH 9.6; Pierce Cat.no. 28382), for 1h at 37°C, washed three times (PBS containing 0.05% Tween-20), and blocked (PBS with 3% BSA) overnight at 4°C–8°C. After a wash step (3x), 100 μL of samples (cell lysates, MxA-positive control cell lysate) were added to the wells and incubated for 2h at room temperature. and 50 μL of the detector antibody (0.5 μg/mL in PBS with 3% BSA) were added to the wells, and the plates were incubated overnight on a plate rotator at 2°C–8°C. Subsequent to washing (4x), the wells were incubated with 100 μL of biotinylated mouse anti-MxA mAb (cone 4E5; Biogen, USA) for 1h at room temperature. After washing (5x), 100uL of streptavidin-poly-HRP (1:10,000 dilution in PBS; Sigma S2438) was added and incubated for 30 min at room temperature, washed (5x), and incubated with 100 μL of tetramethylbenzidine peroxidase substrate peroxidase solution (Sigma T5525, 1 tablet with 9 ml demi-H_2_O, 1 ml 0.5M NaAc, 3 µl H_2_O_2_) for 15 min. in the dark. Color development was monitored, the reaction was stopped (100 μL of 1M H_2_SO_4_) and the optical densities (OD) were measured in a microplate reader (Molecular Devices) at 450 nm. MxA concentrations (ng/ml) were read from a master curve plotted with the standard values using polynomial curve-fitting-software (Softmax Pro). Intra- and inter-assay variation of the ELISA was 7% and 6% respectively.
