# Supplementary material for: The Transcriptome of Paired Major and Minor Salivary Gland Tissue in Patients With Primary Sjögren’s Syndrome
Source: Front Immunol. 2021 Jul 6;12:681941. doi: 10.3389/fimmu.2021.681941 (PMC8291032; doi:10.3389/fimmu.2021.681941)
Supplement: Supplementary file 1 [file DataSheet_1.zip › Supplementary Figures_revised_20210629.docx]

**Supplementary Figures**

**Supplementary Figure 1. Correlation between focus scores and area fraction of CD45+ cells in paired parotid salivary gland (PSG) and labial salivary gland (LSG) samples from pSS patients.** When foci were confluent, a ceiling score of 12 was applied.


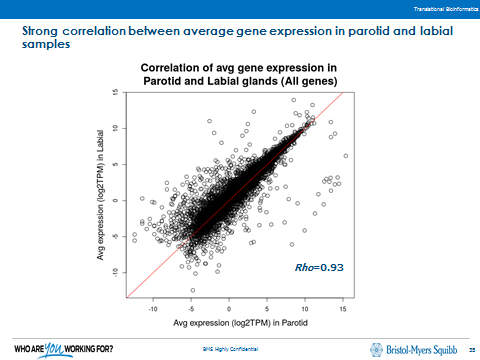


**Supplementary Figure 2. Correlation between average gene expression in parotid and labial gland samples (all genes).**

**
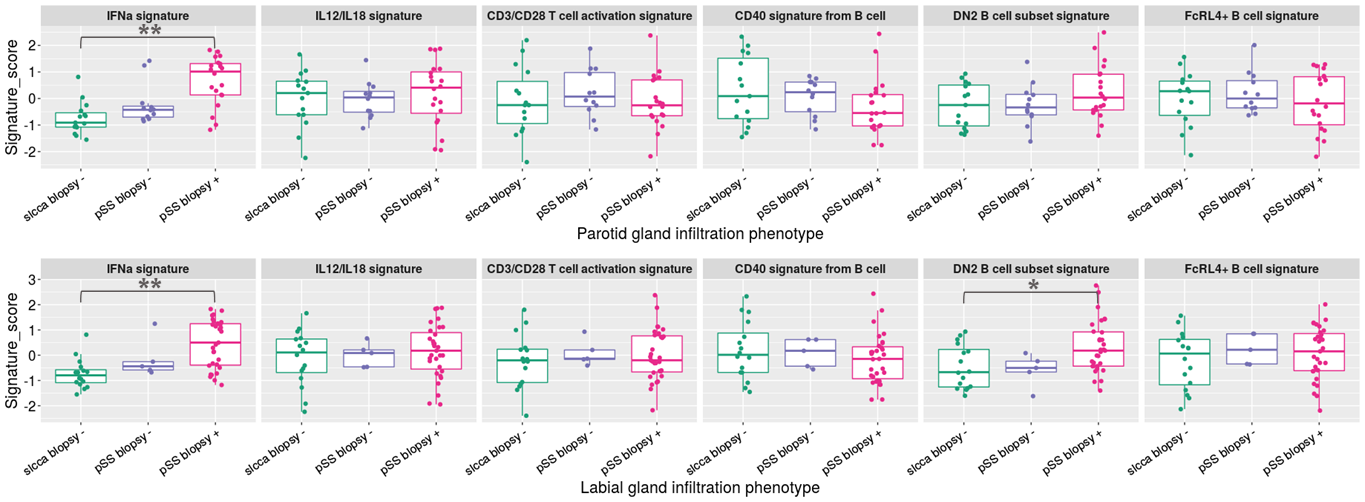
**

**Supplementary Figure 3. Gene signature scores in PBMCs from non-SS sicca and pSS patients.** Gene signature scores were calculated for PBMCs samples. Patients are categorized by the histopathological phenotype of the parotid (upper panel) or labial (lower panel) gland. *: adj.p<0.05; **: adj.p<0.001.

**
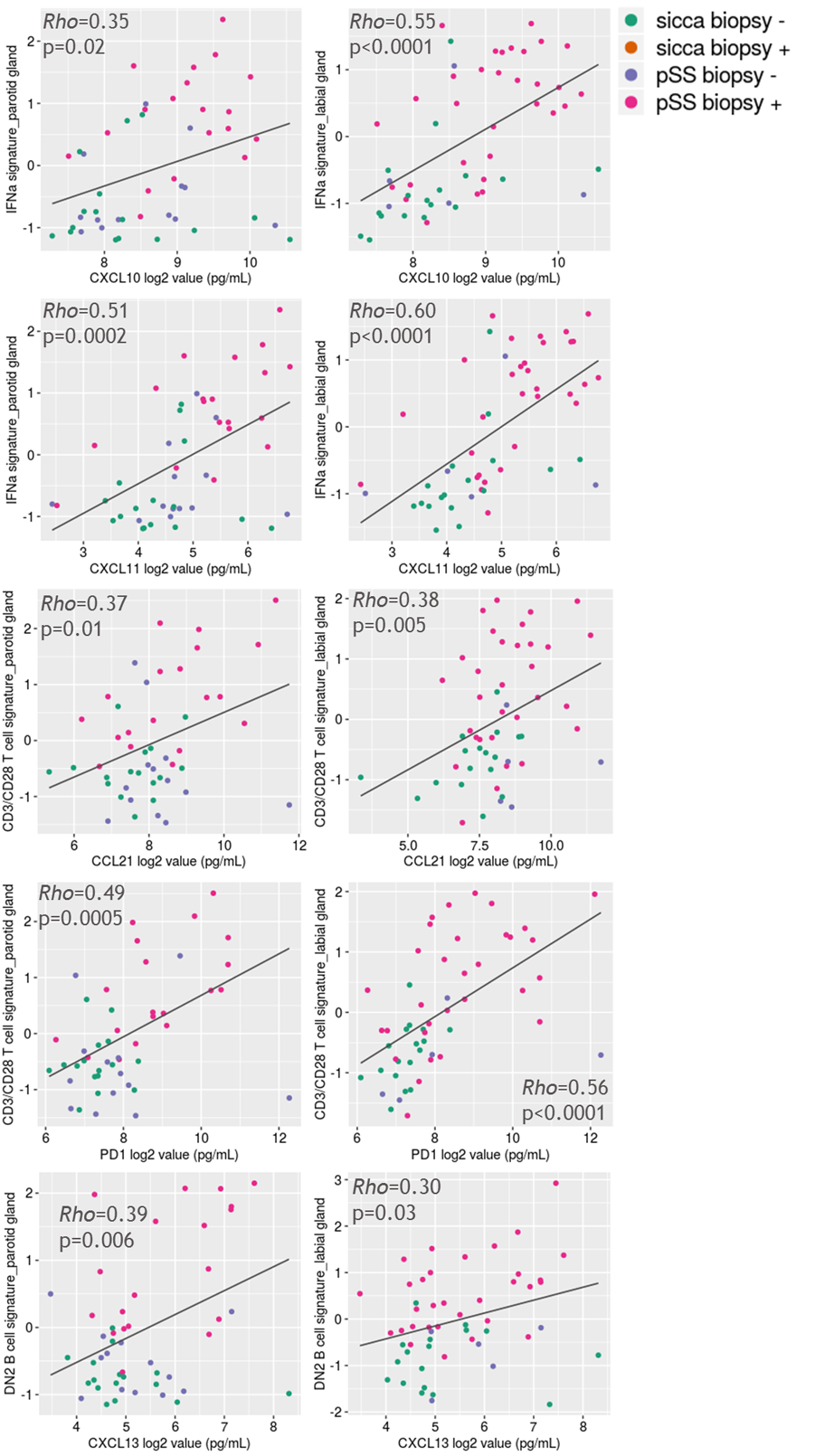
**

**Supplementary Figure 4. Correlations between gene signature scores and cytokine levels in serum.** Serum cytokines that are biologically linked to upregulated gene signatures (e.g., CXCL10 to IFN-α signature) were assessed. Correlations between serum levels of these cytokine and specific gene signature scores in tissue are displayed.
