# Supplementary material for: The Transcriptome of Paired Major and Minor Salivary Gland Tissue in Patients With Primary Sjögren’s Syndrome
Source: Front Immunol. 2021 Jul 6;12:681941. doi: 10.3389/fimmu.2021.681941 (PMC8291032; doi:10.3389/fimmu.2021.681941)
Supplement: Supplementary file 2 [file Table_1.docx]

**Supplementary Table 1. Conditions used to generate gene signatures in vitro**

| **Gene signature** | **Cell types** | **Stimulation time** | **Stimuli** |
| --- | --- | --- | --- |
| IFN-α signature | PBMC | 6h | 5000 U/ml IFN-α A/D (R&D Systems)  1000 U/ml IFN-α+500 nM TYK2i (BMS143848) |
| IL-12/IL-18 signature | PBMC | 6h | 2 ng/ml IL-12 (Peprotech), 5 ng/ml IL-18 (Invitrogen)  5 ng/ml IL-12+10 ng/ml IL-18+500 nM TYK2i (BMS143848) |
| CD3/CD28 T cell signature | Purified T cells | 5h | Anti-CD3 (OKT3, 20 µg/ml), anti-CD28 (clone 9.3, 1 µg/ml) |
| CD40 B cell signature | Purified tonsil B cells | 5h | CD40L trimer (10 µg/ml) |
| CD40 DC signature | Monocyte-derived DCs | 6h | CD40L trimer (10 µg/ml) |
| CD40 monocyte signature | Purified monocytes | 5h | CD40L trimer (10 µg/ml) |
| TLR7 signature | PBMC | 6h | Gardiquimod (3 µg/ml) |
