# Supplementary material for: The Transcriptome of Paired Major and Minor Salivary Gland Tissue in Patients With Primary Sjögren’s Syndrome
Source: Front Immunol. 2021 Jul 6;12:681941. doi: 10.3389/fimmu.2021.681941 (PMC8291032; doi:10.3389/fimmu.2021.681941)
Supplement: Supplementary file 6 [file Table_5.docx]

**Supplementary Table 5**

|  | Parotid |  |  |  | Labial |  |  |  |
| --- | --- | --- | --- | --- | --- | --- | --- | --- |
|  | **Group I** | **Group**  **III** | **Group**  **IV** | **P-value** | **Group**  **I** | **Group**  **III** | **Group**  **IV** | **P-value** |
| UWSF (mL/min) | 0.13 (0.00-0.50) | 0.04 (0.00-0.60) | 0.09 (0.00-0.68) | 0.608^a^ | 0.16 (0.00-0.58) | 0.01 (0.01-0.14) | 0.09 (0.00-0.68) | 0.127^a^ |
| SWSF (mL/min) | 0.65 (0.08-1.80) | 0.67 (0.01-2.33) | 0.66 (0.07-1.65) | 0.998^a^ | 0.73 (0.08-2.41) | 0.55 (0.18-0.77) | 0.66 (0.00-2.33) | 0.297^a^ |

^a^ Kruskal Wallis test. UWSF: Unstimulated whole salivary flow; SWSF: Stimulated whole salivary flow (by chewing). Group-I: Non-SS sicca patient with negative biopsy. Group-III: pSS patients with negative biopsy. Group-IV: pSS patients with positive biopsy. Results are presented as median (range).
